# Comparison of Anti-Oxidative Effect of Human Adipose- and Amniotic Membrane-Derived Mesenchymal Stem Cell Conditioned Medium on Mouse Preimplantation Embryo Development

**DOI:** 10.3390/antiox10020268

**Published:** 2021-02-09

**Authors:** Kihae Ra, Hyun Ju Oh, Eun Young Kim, Sung Keun Kang, Jeong Chan Ra, Eui Hyun Kim, Se Chang Park, Byeong Chun Lee

**Affiliations:** 1 Department of Theriogenology and Biotechnology, College of Veterinary Medicine, Seoul National University, Seoul 08826, Korea; ragh1102@snu.ac.kr (K.R.); ohj@mkbiotech.co.kr (H.J.O.); hyun9214@snu.ac.kr (E.H.K.); 2 Research and Development Center, MKbiotech Co., Ltd., 99 Daehak-ro, Daejeon 34134, Korea; 3 Biostar Stem Cell Research Institute, R Bio Co., Ltd., Seoul 08506, Korea; naraokke@stemcellbio.com (E.Y.K.); kangsk@stemcellbio.com (S.K.K.); jcra@stemcellbio.com (J.C.R.); 4 Laboratory of Aquatic Biomedicine, College of Veterinary Medicine, Seoul National University, Seoul 08826, Korea

**Keywords:** adipose-derived mesenchymal stem cell, amniotic membrane-derived mesenchymal stem cell, antioxidants, assisted reproductive technology, conditioned medium, embryo, in vitro culture, in vitro fertilization, oxidative stress

## Abstract

Oxidative stress is a major cause of damage to the quantity and quality of embryos produced in vitro. Antioxidants are usually supplemented to protect embryos from the suboptimal in vitro culture (IVC) environment. Amniotic membrane-derived mesenchymal stem cells (AMSC) have emerged as a promising regenerative therapy, and their paracrine factors with anti-oxidative effects are present in AMSC conditioned medium (CM). We examined the anti-oxidative potential of human AMSC-CM treatment during IVC on mouse preimplantation embryo development and antioxidant gene expression in the forkhead box O (FoxO) pathway. AMSC-CM (10%) was optimal for overall preimplantation embryo developmental processes and upregulated the expression of FoxOs and their downstream antioxidants in blastocysts (BL). Subsequently, compared to adipose-derived mesenchymal stem cell (ASC)-CM, AMSC-CM enhanced antioxidant gene expression and intracellular GSH levels in the BL. Total antioxidant capacity and SOD activity were greater in AMSC-CM than in ASC-CM. Furthermore, SOD and catalase were more active in culture medium supplemented with AMSC-CM than in ASC-CM. Lastly, the anti-apoptotic effect of AMSC-CM was observed with the regulation of apoptosis-related genes and mitochondrial membrane potential in BL. In conclusion, the present study established AMSC-CM treatment at an optimal concentration as a novel antioxidant intervention for assisted reproduction.

## 1. Introduction

The success rate of assisted reproductive technologies (ART) to surmount infertility has increased with the improvement of conditions for embryo in vitro production [1]. The balance of reactive oxygen species (ROS) and antioxidants is maintained at physiologically normal levels in female reproductive systems, but is disrupted in vitro, resulting in an increase in exposure to oxidative damage risk [2]. In the process of assisted reproduction, a number of external factors causing oxidative stress appear from technical procedures to environmental sources [3]. Subsequently, oxidative stress due to accumulated ROS in in vitro-produced embryos impairs the efficiency of embryonic development and induces reproductive failure due to an increase in embryo fragmentation and apoptosis, and a decrease in fertilization rate and blastocyst (BL) development [4,5,6]. Accordingly, the application of antioxidants to ART can be an effective intervention to counteract the oxidative damage in in vitro-produced embryos [7], especially to improve the in vitro culture (IVC) medium for favorable outcomes in preimplantation embryo development. Accumulating studies have indicated that the addition of antioxidants to IVC medium improves preimplantation embryo development by regulating the embryonic environment and protecting embryos from oxidative damage [8,9,10,11], which leads to the decrease in developmental competence of embryos produced in vitro compared to that of embryos developed in vivo [12].

Mesenchymal stem cells (MSCs) are known to ameliorate oxidative stress through the upregulation of enzymatic antioxidants [13]. MSCs isolated from multiple tissue sources have common but various features, which emphasize their importance as regenerative medicine [14]. Among multiple sources, adipose-derived MSCs (ASCs) are the most widely studied, forming the basis of research on MSC-based therapy [15] and is representatively known a strong antioxidant [6,13,16,17,18]. Contemporarily, amniotic membrane-derived MSCs (AMSC) have emerged as a novel candidate in the field of regenerative medicine because of their unique advantages, including noninvasive isolation, stable immunogenicity, abundant availability, multipotency for all three germ layers, and no associated ethical issues [19]. The amniotic membrane is a constituent of the placenta with its essential function of nutrient supplementation and physical protection for the fetus during pregnancy, but is generally discarded post-partum and infrequently utilized compared to other MSCs [20]. However, AMSCs retain the anti-microbial, anti-tumorigenic, immunomodulatory, and anti-inflammatory characteristics of amniotic membrane [21]. Recent studies on potential therapeutic features of human AMSCs have focused on their role in immunomodulation, suppressing inflammation, and inhibiting oxidative damage [22,23,24,25]. Although diverse studies support the anti-oxidative effect of AMSCs in diseases models, the therapeutic applications of AMSCs were restricted to cell transplantation [22,26,27]. The regenerative effect of stem cell therapy is mainly facilitated by its paracrine factors, such as cytokines and growth factors, rather than by direct regenerative mechanisms [28,29]. These stem cell-derived paracrine factors are secreted during cell culture and are present in the stem cell-conditioned medium (CM) [30]. The therapeutic efficacy of the CM is comparable to that of the conventional cell-based therapy. Furthermore, the use of CM offers several advantages over conventional stem cell-therapy, such as improved reproducibility, no requirement to match the donors and recipients, and no risk of immune rejection [31]. 

In the present study, we aimed to investigate the anti-oxidative potential of AMSC-CM and establish the optimal concentration for AMSC-CM treatment during embryo IVC. Consequently, the anti-oxidative and anti-apoptotic effects of AMSC-CM were evaluated as compared to ASC-CM in the development of mouse preimplantation embryos.

## 2. Materials and Methods

### 2.1. Chemicals and Reagents

All materials were purchased from Sigma-Aldrich (St. Louis, MO, USA), unless otherwise specified.

### 2.2. Culture and Characterization of ASCs and AMSCs

Both ASCs and AMSCs were obtained from R Bio Stem Cell Research Center under GMP conditions. All subjects gave their informed consent for inclusion before they participated in the study. The protocol was approved by the Ethics Committee of Biostar Stem Cell Technology (IRB NO. 2019-03). ASCs were cultured and characterized as previously described [6]. For the establishment of AMSCs, cryopreserved AMSCs (1 × 10^6^) from the amnion tissue of three female donors were cultured in T-175 flasks containing RPME-P (R BIO, Seoul, Korea) supplemented with 1% antibiotic-antimycotic solution at 37 °C with 5% CO_2_. The AMSCs were cultured in AMSC medium (R BIO) until 80–90% confluency and non-adherent cells were discarded through medium change. The immunophenotypic markers of cultured AMSCs was characterized by flow cytometry. AMSCs (1 × 10^6^) were suspended in phosphate-buffered saline (PBS) and labeled with fluorescein isothiocyanate and phycoerythrin isotype controls. The labeled cells were incubated for 30–60 min with the following antibodies against human antigens: MSC positive markers (CD73, CD90, CD105, CD29, and CD44) and negative markers (CD31, CD34, and CD45) (BD Biosciences, San Jose, CA, USA). After the cells were washed with PBS, the analysis was conducted with FACSCalibur™ flow cytometer (BD Biosciences) and CellQuest Pro software (BD Biosciences). 

### 2.3. Preparation of ASC-CM and AMSC-CM

ASC-CM were collected using the same method as previously described [6]. AMSCs per donor (passage 6) were cultured in RPME-P until 90% confluency, and then the medium was replaced to Dulbecco’s modified Eagle’s medium (DMEM) after washing twice with PBS. The culture medium was collected every 24 h and then DMEM was added to the original flask. Supernatants were collected for 5 days and then pooled. To obtain CM, the pooled supernatant was centrifuged (1700 rpm, 5 min) and then filtered in a 0.22-µm filter. Lastly, filtered CM of donors was equally mixed and concentrated 10× by centrifugation at 3000× *g* for 90 min using a filter tube (Vivaspin 20, GE healthcare, Chicago, IL, USA).

### 2.4. Experimental Animals

All experiments using experimental animals in this study were approved by the Institutional Animal Care and Use Committee of Seoul National University (SNU-170511-2-4). Seven-week-old female and 10-week-old male ICR mice were purchased from Orient Bio (Gapyeong, Korea). Mice were kept in an animal facility under conventional environment with the light/dark cycle, humidity and temperature regulated.

### 2.5. In Vitro Fertilization and Culture

After cervical dislocation of mature male mice, caudal epididymides were removed and the duct of the caudal epididymis was incised. The sperm stored inside were dispersed into a droplet of CARD medium (Cosmo Bio Co., Tokyo, Japan). Sperm were incubated for an hour at 36 °C to enable capacitation. The induction of superovulation of mature female mice was conducted by an intraperitoneal injection of 10 IU pregnant mare serum gonadotropin and human chorionic gonadotropin (hCG) after 47 h. The cumulus-oocyte complexes (COCs) were recovered from the oviductal ampulla of the mice 16 h after hCG injection and transferred to a droplet of CARD medium. The sperm suspension was treated with COCs for insemination and incubated for 3 h at 36 °C. In vitro fertilized embryos were washed and cultured in fresh human tubal fluid (Cosmo Bio Co., Tokyo, Japan) at 36 °C for 24 h. Embryos that cleaved to the 2- or 4-cell were randomly divided and then cultured in the groups as described in experimental design for 96 h. The embryo development was evaluated by assessing the number of 4-cell, 16-cell, BL, and hatched BL using a stereomicroscope. The temperature was set based on the literature and our preliminary study. The literature demonstrated that slightly lower temperature could be physiologically relevant to reproductive tissues [32,33,34] and comparable to traditional 37 °C for reproductive outcomes [35,36,37]. In our preliminary study, developmental rate to BL at 36 °C showed no difference to that at 37 °C, and both rates were observed within the normal range.

### 2.6. Experimental Design

First, fertilized embryos were cultured in continuous single culture-NX (CSCM-NX; FUJIFILM Irvine Scientific, Santa Ana, CA, USA) containing 10%, 20%, and 50% (*v*/*v*) AMSC-CM. After determining the optimal concentration of AMSC-CM for IVC supplementation, embryos were cultured followed by in vitro fertilization in CSCM-NX containing ASC-CM or AMSC-CM. The optimal concentration of ASC-CM was set 5% (*v*/*v*) as previously reported [6]. The control group was cultured in CSCM-NX medium without CM supplementation.

### 2.7. Quantitative Reverse Transcription-Polymerase Chain Reaction (qRT-PCR)

RNA was extracted from BLs using an RNAqueous™-Micro Total RNA Isolation Kit (Ambion, Austin, TX, USA), according to the manufacturer’s instructions. The concentration of extracted total RNA was quantified by a NanoDrop 2000 Spectrophotometer (Thermo Fisher Scientific, Wilmington, DE, USA) and presented in Table S1. Using the RNA, complementary DNA (cDNA) was synthesized by a Maxime RT premix kit (iNtRON, Gyeonggi, Korea). qRT-PCR was carried out using a StepOnePlus Real-Time PCR System (Applied Biosystems, Foster City, CA, USA) and the protocol in detail was previously described [18]. The expression of target genes was measured and normalized relative to the control house-keeping gene, 18S rRNA [38,39,40]. The gene expression values were calculated as previously described [18]. The list of primers is presented in Table 1.

### 2.8. Intracellular ROS and Glutathione (GSH) Detection

The levels of intracellular ROS and GSH were measured in BLs from the control, ASC-CM, and AMSC-CM groups by staining respectively with H_2_DCFDA (2,7′-dichlorodihydrofluorescein diacetate) and CellTracker Blue (4-chloromethyl-6,8-difluoro-7-hydroxycoumarin; CMF_2_HC). BLs from each group were incubated in 1% polyvinyl alcohol (PVA)-PBS containing 10 μM H_2_DCFDA or CellTracker Blue in the dark at 25 °C. After 30 min, BLs were washed and moved to a droplet of PVA-PBS covered with mineral oil. The quantitative intensity of fluorescence was evaluated under an epifluorescence microscope (TE2000-S; Nikon, Tokyo, Japan) using filters (ROS: 460 nm, GSH: 370 nm) and analyzed by Image J software version 1.52 (National Institutes of Health, Bethesda, MO, USA).

### 2.9. Antioxidant Capacity and Enzyme Activity Assays

The total antioxidant capacity (TAC), superoxide dismutase (SOD), and catalase activity were measured using OxiSelect™ assay kits (Cell Biolabs Inc., San Diego, CA, USA) according to the manufacturer’s protocol. Non-conditioned medium as control, ASC-CM and AMSC-CM were assessed for TAC, SOD, and CAT activity levels. Culture medium of the control, ASC-CM, and AMSC-CM groups before and after IVC were assessed for SOD and CAT activity levels. The results of each colorimetric assay were assessed using measured absorbances at 490 nm for TAC and SOD activity, and 520 nm for CAT activity.

### 2.10. Mitochondrial Membrane Potential Assay

BLs from the control, ASC-CM, and AMSC-CM groups were washed in 1% PVA-PBS and fixed in 4% paraformaldehyde-PBS for 1 h. After washing in 1% PVA-PBS, BLs were incubated in 1% PVA-PBS containing 2 μL JC-1 solution (Abcam, Cambridge, UK) and then washed in fresh 1% PVA-PBS. After 30 min, BLs were placed on a droplet of glycerol on a microscope glass slide with a coverslip. The fluorescence intensity of JC-1 aggregate at 590 nm and JC-1 monomer at 530 nm was evaluated using epifluorescence microscope and analyzed using Image J software version 1.52.

### 2.11. Statistical Analysis

A Kolmogorov–Smirnov test was conducted as normality test. Unpaired *t*-test was used to compare two groups. One-way ANOVA followed by Newman–Keuls or Tukey’s post-hoc test and two-way ANOVA test with Bonferroni post-test were used to compare more than two groups. GraphPad Prism version 5 (GraphPad Software, San Diego, CA, USA) was used for statistical analyses. Data are presented as mean ± standard error of the mean (SEM), and a *p*-value < 0.05 was considered statistically significant among the groups. All experiments were performed with at least three replicates.

## 3. Results

### 3.1. Characterization of AMSC and ASC

**Figure 1:**
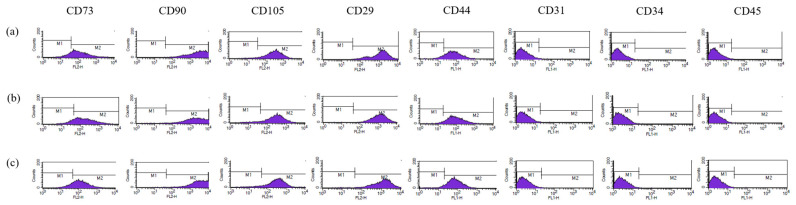
Characterization of human amniotic membrane-derived mesenchymal stem cell (AMSC). AMSCs isolated from three donors (**a**–**c**) were positive for CD73, CD90, CD105, CD29, CD44, and negative for CD31, CD34, and CD45.

AMSCs were analyzed with flow cytometry to identify the expression of phenotypic markers (Figure 1) and confirmed that AMSCs from all donors were positive for mesenchymal markers (CD73, CD90, CD105, CD29, and CD44), and negative for the endothelial marker (CD31) and hematopoietic markers (CD34 and CD45). The result of ASC characterization was previously described [6].

### 3.2. Effects of Various Concentrations of AMSC-CM on Embryo Development

Embryo development to 4-, 16-cell stages, BL, and hatched BL was evaluated to determine the optimal concentration of AMSC-CM supplementation among the three different concentrations of AMSC-CM (10%, 20%, and 50%). As shown in Table 2, embryo development rate to the 4-cell stage was significantly lower in the 50% AMSC-CM group (79.6 ± 4.0) than in the control (91.8 ± 1.8, *p* < 0.05), 10% (92.5 ± 4.8, *p* < 0.05), and 20% AMSC-CM (93.4 ± 3.0, *p* < 0.05) groups. Moreover, the embryo development rate to the 16-cell stage was significantly higher in the 10% AMSC-CM group (74.3 ± 4.8) than in the 20% (61.2 ± 3.5, *p* < 0.05) and 50% AMSC-CM (59.5 ± 3.8, *p* < 0.05) groups. The rate of BL formation in the 10% AMSC-CM group (51.7 ± 4.1) was significantly higher than that in the control (38.6 ± 4.5, *p* < 0.05), 20% (30.7 ± 5.5, *p* < 0.05), and 50% AMSC-CM (28.3 ± 6.8, *p* < 0.05) groups. BL hatching rate in the 10% AMSC-CM group (32.6 ± 5.9) was also significantly higher than that in the control (19.4 ± 4.6, *p* < 0.05), 20% (19.1 ± 3.5, *p* < 0.05), and 50% AMSC-CM (18.8 ± 3.4, *p* < 0.05) groups.

### 3.3. Comparison of the Effects of ASC-CM and AMSC-CM on Embryo Development

Following the former experiment, which confirmed 10% as the optimal concentration of AMSC-CM treatment, the effects of ASC-CM and AMSC-CM during IVC on embryo development to the 4-, 16-cell stages, BL, and hatched BL were compared. As presented in Table 3, the developmental rate of embryos to the 4-cell stage was similar among groups, but the rate to the 16-cell stage was significantly increased in the AMSC-CM group (87.6 ± 5.1) compared to the control group (73.7 ± 3.3, *p* < 0.05). In addition, BL formation rate of AMSC-CM group (65.7 ± 3.3) was significantly higher than that of the control group (44.4 ± 5.2, *p* < 0.05). The developmental rates of 16-cell and BL in the ASC-CM groups (79.2 ± 4.0 and 56.4 ± 2.8, respectively) showed no difference from those of the other groups. The rate of hatched BL in the AMSC-CM group was greater than that in the other groups, although the difference was not statistically significant. 

### 3.4. Comparative Effects of ASC-CM and AMSC-CM on Antioxidant Gene Expression in BL

**Figure 2:**
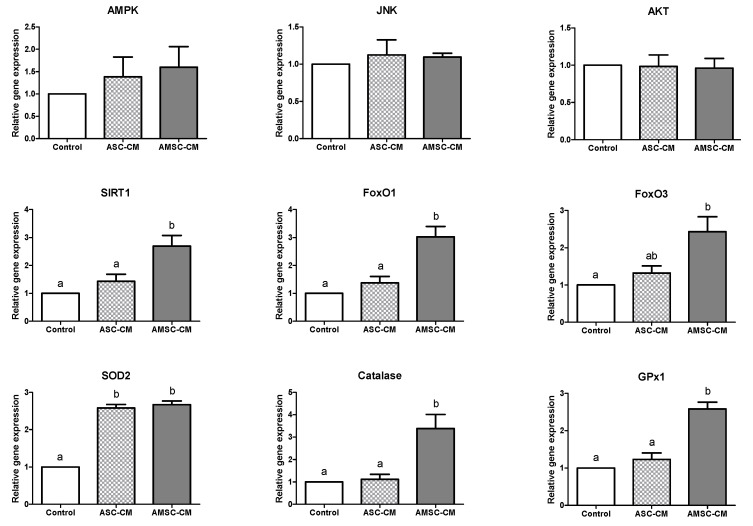
Relative antioxidant gene expression in blastocysts cultured in control, human adipose-derived mesenchymal stem cell conditioned medium (ASC-CM) and amniotic membrane-derived mesenchymal stem cell conditioned medium (AMSC-CM). Data are normalized to housekeeping gene 18S rRNA and presented as mean ± standard error of the mean (SEM). Superscript letters in each column indicate significant differences (*p* < 0.05).

BLs developed in the control, ASC-CM, and AMSC-CM groups were analyzed for the expression of the antioxidant genes in the forkhead box O (FoxO) pathway and apoptosis-related genes, as presented in Figure 2. First, the expression of upstream regulators of FoxO was evaluated; the expression of AMP-activated protein kinase (AMPK), c-Jun N-terminal kinase (JNK), and protein kinase B (AKT) exhibited no significant differences among groups. Next, the level of sirtuin (SIRT) 1, a mediator of FoxO, was shown to be significantly higher in the AMSC-CM group (2.7 ± 0.4) than in the control (1.0 ± 0.0, *p* < 0.05) and ASC-CM group (1.4 ± 0.3, *p* < 0.05). FoxO1 and FoxO3 levels were significantly increased in the AMSC-CM group (3.0 ± 0.4 and 2.4 ± 0.4, respectively) compared to the control group (1.0 ± 0.0, *p* < 0.05). Furthermore, FoxO1 expression in the AMSC-CM group (3.0 ± 0.4) was significantly higher than that in the ASC-CM group (1.4 ± 0.2, *p* < 0.05) but FoxO3 levels were similar between two groups. The level of SOD2 was significantly greater in the ASC-CM and AMSC-CM groups (2.6 ± 0.1 and 2.7 ± 0.1, respectively) than in the control group (1.0 ± 0.0, *p* < 0.05). Catalase and glutathione peroxidase (GPx) 1 levels were significantly increased in the AMSC-CM group (3.4 ± 0.6 and 2.6 ± 0.2, respectively) compared to the control (1.0 ± 0.0, *p* < 0.05) and ASC-CM groups (1.1 ± 0.2 and 1.2 ± 0.2, respectively, *p* < 0.05).

### 3.5. Comparative Effects of ASC-CM and AMSC-CM on Intracellular Oxidative Stress in BL

**Figure 3:**
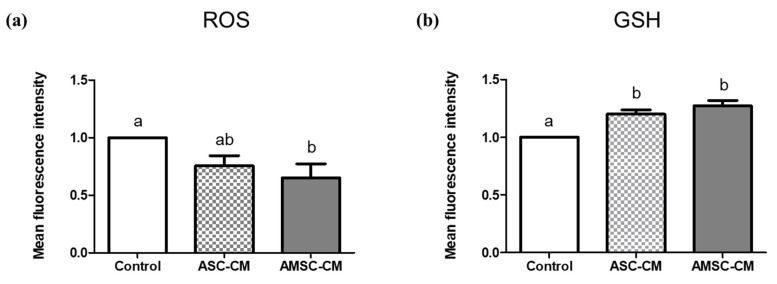
Evaluation of intracellular reactive oxygen species (ROS) and glutathione (GSH) in blastocysts (BL) cultured with human adipose-derived mesenchymal stem cell conditioned medium (ASC-CM) and amniotic membrane-derived mesenchymal stem cell conditioned medium (AMSC-CM). (**a**) ROS and (**b**) GSH level in BL. Data are presented as the mean ± SEM. Superscript letters in each column indicate significant differences (*p* < 0.05).

Anti-oxidative effects of ASC-CM and AMSC-CM were evaluated through the measurement of ROS and GSH in BL. ROS levels in the BL of the AMSC-CM group (0.7 ± 0.1) were significantly lower than those in the control group (1.0 ± 0.0, *p* < 0.05), but the ASC-CM group showed no significant difference with the other groups (Figure 3a). As shown in Figure 3b, GSH levels in the BL in both ASC-CM (1.2 ± 0.0) and AMSC-CM (1.3 ± 0.0) groups were significantly increased compared to the control group (1.0 ± 0.0, *p* < 0.05).

### 3.6. Comparison of Antioxidant Biomarkers in ASC-CM and AMSC-CM

**Figure 4:**
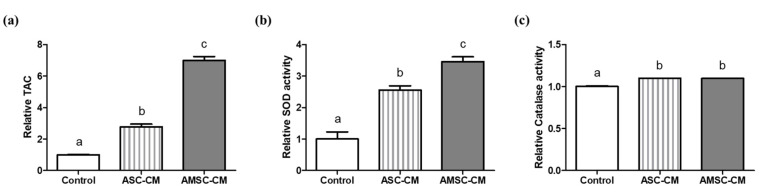
Comparison of antioxidant biomarkers level in human adipose-derived mesenchymal stem cell conditioned medium (ASC-CM) and amniotic membrane-derived mesenchymal stem cell conditioned medium (AMSC-CM). (**a**) Total antioxidant capacity (TAC), (**b**) superoxide dismutase (SOD) activity, and (**c**) catalase activity. Data are normalized to average value of control and presented as the mean ± SEM. Superscript letters in each column indicate significant differences (*p* < 0.05).

TAC and SOD activity of both ASC-CM (2.8 ± 0.2 and 2.6 ± 0.1) and AMSC-CM (7.0 ± 0.2 and 3.5 ± 0.2) were significantly higher when compared to the control (1.0 ± 0.0 and 1.0 ± 0.2, respectively, *p* < 0.05, Figure 4a,b). Comparing ASC-CM and AMSC-CM, TAC and SOD activity of AMSC-CM was significantly greater than ASC-CM (*p* < 0.05, Figure 4a,b). Catalase activities in ASC-CM (1.1 ± 0.0) and AMSC-CM (1.1 ± 0.0) were similar but significantly higher than the control (1.0 ± 0.0, *p* < 0.05, Figure 4c).

### 3.7. Comparison of Antioxidant Biomarkers in Culture Medium with ASC-CM and AMSC-CM

**Figure 5:**
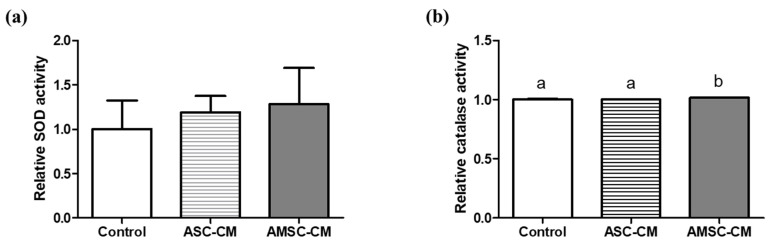
Comparison of antioxidant biomarkers level in fresh culture medium supplemented with human adipose-derived mesenchymal stem cell conditioned medium (ASC-CM) and amniotic membrane-derived mesenchymal stem cell conditioned medium (AMSC-CM). (**a**) Superoxide dismutase (SOD) and (**b**) catalase activity. Data are normalized to average value of control and presented as the mean ± SEM. Superscript letters in each column indicate significant differences (*p* < 0.05).

**Figure 6:**
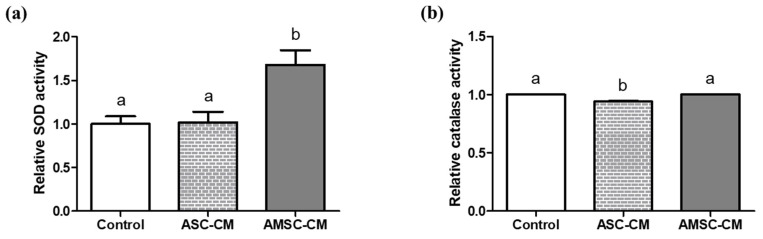
Comparison of antioxidant biomarker levels in culture medium supplemented with human adipose-derived mesenchymal stem cell conditioned medium (ASC-CM) and amniotic membrane-derived mesenchymal stem cell conditioned medium (AMSC-CM) which was collected after 5 days of embryo culture. (**a**) Superoxide dismutase (SOD) and (**b**) catalase activity. Data are normalized to average value of control and presented as the mean ± SEM. Superscript letters in each column indicate significant differences (*p* < 0.05).

Catalase activity was significantly increased in pre-IVC medium supplemented with AMSC-CM (1.02 ± 0.0) compared to the control (1.0 ± 0.0, *p* < 0.05) and ASC-CM (1.0 ± 0.0, *p* < 0.05, Figure 5b). Likewise, SOD activity in post-IVC medium supplemented with AMSC-CM (1.7 ± 0.2) was significantly greater than that in the control and ASC-CM groups (1.0 ± 0.1 and 1.0 ± 0.1, respectively, *p* < 0.05, Figure 6a). Furthermore, catalase activity was significantly higher in post-IVC medium supplemented with AMSC-CM (1.0 ± 0.0) than in ASC-CM (0.9 ± 0.0, *p* < 0.05, Figure 6b) but not when compared to the control.

### 3.8. Comparative Effects of ASC-CM and AMSC-CM on Apoptosis-Related Gene Expression in BL

**Figure 7:**
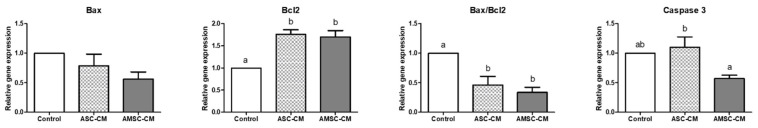
Relative apoptosis-related gene expression in blastocysts cultured in the control, human adipose-derived mesenchymal stem cell conditioned medium (ASC-CM) and amniotic membrane-derived mesenchymal stem cell conditioned medium (AMSC-CM) group. Data are normalized to housekeeping gene 18S rRNA and presented as mean ± standard error of the mean (SEM). Superscript letters in each column indicate significant differences (*p* < 0.05).

To assess not only oxidative stress but also the consequent apoptosis of BL, the relative expression of the anti-apoptotic and pro-apoptotic genes was analyzed. The expression levels of B cell leukemia/lymphoma 2 (Bcl2) in both ASC-CM (1.8 ± 0.1) and AMSC-CM (1.7 ± 0.1) groups were significantly greater than those of the control (1.0 ± 0.0, *p* < 0.05, Figure 7). The ratio of Bcl2-associated X (Bax) to Bcl2 expression level in both ASC-CM (0.5 ± 0.1) and AMSC-CM (0.3 ± 0.1) groups was significantly lower than those of the control (1.0 ± 0.0, *p* < 0.05, Figure 7). Although no differences were found between the relative gene expression level of Bax among groups, Caspase 3 levels were significantly lower in the AMSC-CM group (0.6 ± 0.1) than in the ASC-CM group (1.1 ± 0.2, *p* < 0.05, Figure 7). 

### 3.9. Comparative Effects of ASC-CM and AMSC-CM on Intracellular Apoptosis in BL

**Figure 8:**
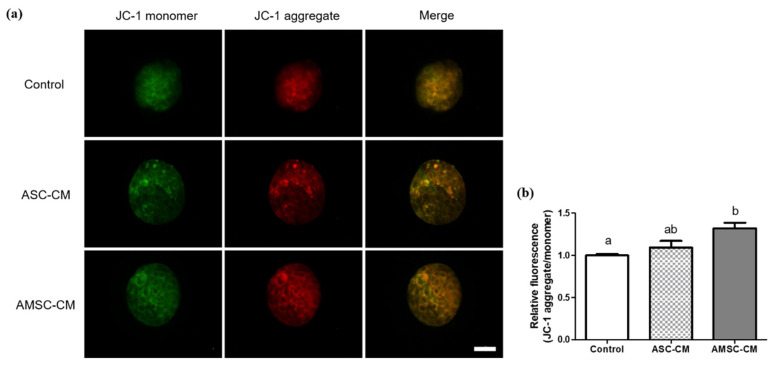
Assessment of mitochondrial membrane potential in blastocysts (BL) cultured with human adipose-derived mesenchymal stem cell conditioned medium (ASC-CM) and amniotic membrane-derived mesenchymal stem cell conditioned medium (AMSC-CM). (**a**) Representative fluorescent images of JC-1 monomer (green) and aggregate (red) stained BL. Original magnification 400×. Scale bar = 50 μm. (**b**) The ratio of JC-1 aggregate (red) to JC-1 monomer (green) presented by quantifying fluorescence intensity of JC-1 mitochondrial membrane potentials in BL. Data are expressed as the mean ± standard error of the mean (SEM). Superscript letters in each column indicate significant differences (*p* < 0.05).

Mitochondrial membrane potential was visualized and measured as an indicator of intracellular apoptosis using JC-1 fluorescence staining of BLs from the control, ASC-CM, and AMSC-CM groups (Figure 8a). The ratio of JC-1 aggregate to JC-1 monomer in BLs of the AMSC-CM group (1.3 ± 0.1) was significantly higher than that of the control group (1.0 ± 0.0, *p* < 0.05). However, the ratio in BLs from the ASC-CM group was similar to that of the other groups (Figure 8b).

## 4. Discussion

The present study was conducted with the purpose of (1) examining the anti-oxidative effect of human AMSC-CM treatment during IVC on mouse preimplantation embryo development, while simultaneously evaluating antioxidant gene expression, more specifically, the genes in the FoxO pathway, and (2) comparing human ASC-CM and AMSC-CM as supplementation for mouse embryo culture with regard to their anti-oxidative and anti-apoptotic effects.

At first, the effect of various concentrations of AMSC-CM treatment (10%, 20%, and 50%) during IVC was investigated to establish the optimal concentration for the development of in vitro fertilized mouse embryos. The rate of embryos that developed to the 4-cell and 16-cell stage were significantly lower in the 50% AMSC-CM group than in the 10% AMSC-CM group. These results indicate that a high CM concentration does not ensure better efficiency of embryo development regardless of the large quantity of cytokines as previously explained [6]. Remarkably, we found that 10% AMSC-CM significantly improved BL formation rate (Table 2), which is an index for embryo developmental potential and consequently determines the success of implantation [41], compared to the control group as well as the other higher concentration of AMSC-CM treated groups. In addition, the 10% AMSC-CM group showed the most enhanced BL hatching ability, a crucial precondition for successful implantation and pregnancy rates [42]. Together, our results indicated that 10% AMSC-CM treatment during IVC is optimal for overall preimplantation embryo developmental processes from early cleavage to BL hatching. 

A few studies have compared the characteristics and proliferation rate of ASC and AMSC [43,44,45], but to the best of our knowledge, the comparison between anti-oxidative effects of ASC-CM and AMSC-CM has never been reported, particularly on the embryo and its culture medium. Therefore, the effects of ASC-CM and AMSC-CM treatment at the respective confirmed optimal concentrations in embryo IVC medium were compared. As validated by the results presented in Table 2, AMSC-CM treatment improved embryo development compared to the control. The embryo developmental rate of the AMSC-CM group was greater than that of the ASC-CM group at all the assessed stages (Table 3), but the difference was not statistically significant. The expression of antioxidant genes in the FoxO signaling pathway was analyzed to evaluate the quality of BLs cultured with ASC-CM or AMSC-CM. FoxO transcription factors modulate various cellular functions, including differentiation, growth, metabolism, and apoptosis [46]. These factors predominantly regulate the oxidative stress response by controlling the expression of manganese-dependent SOD (SOD2), catalase, and GPx1 that constitute the primary defense mechanism against ROS [47]. FoxO is considered as a therapeutic target for infertility and is critical for the preimplantation embryo development in mice [46]. Specifically, among the mammalian FoxO family, FoxO1 and FoxO3 are key players in female reproductive processes [48]. We found that the relative expression of FoxO1 and FoxO3 was significantly increased in the BLs cultured with AMSC-CM as compared to those of the control group. Furthermore, the expression levels of SOD2, catalase, and GPx1, downstream targets of the FoxO subfamily, were significantly greater in the AMSC-CM group than in the control group (Figure 2). Remarkably, compared with ASC-CM, AMSC-CM promoted the expression of FoxO1, catalase, and GPx1 (Figure 2). We also analyzed genes that function as upstream regulators of FoxO such as AMPK [49] and JNK [50], as well as AKT [51], but none of the genes exhibited notable differences in expression, which seems to have an ambiguous influence on FoxO activity in that FoxO receives various signals from growth factors, metabolic and oxidative stress [52] and involves numerous mechanisms for its regulation [53]. However, we found an increase in SIRT1 expression in BL cultured with AMSC-CM, which is a crucial regulator of oxidative stress that protects cells by upregulating antioxidant activity through FoxO-dependent mechanisms and, in particular, the interaction of SIRT1 and FOXO3a mainly functions in protecting oocytes against loss of developmental competence from reproductive aging [54]. GSH is a representative non-enzymatic antioxidant that is essential for embryo development after fertilization up to the BL stage [55]. In this study, intracellular GSH levels were increased in BLs with ASC-CM and AMSC-CM treatment, but ROS levels were decreased only in the AMSC-CM group (Figure 3). ROS are attenuated by a collaborative defense system comprising enzymatic and non-enzymatic antioxidants [56]. Collectively, the results described above suggest that AMSC-CM exerts an anti-oxidative effect during embryo culture by improving the expression of both enzymatic and non-enzymatic antioxidants in BL.

We then investigated the antioxidant biomarker activity in CM, pre- and post-IVC medium containing different CM. In addition to TAC, a complex indicator showing the comprehensive activity of various antioxidants [57], the activities of SOD and catalase were all greater in both ASC-CM and AMSC-CM when compared to non-conditioned medium (Figure 4). Notably, we found evident difference that AMSC-CM showed greater level of antioxidant biomarkers than ASC-CM. The results are consistent with various studies that reported the factors secreted from MSC contain antioxidants as one of the predominant elements, which are included in CM and exert anti-oxidative effects in paracrine mechanisms [13,58,59,60]. In particular, numerous growth factors found in CM of human amnion tissue and AMSC [61] have been identified to function as antioxidants including insulin-like growth factor [62], platelet-derived growth factor [63], epidermal growth factor [64], hepatocyte growth factor [65] and fibroblast growth factor [66]. Moreover, pre- and post-IVC medium analysis revealed that the activities of SOD and catalase were higher in culture medium supplemented with AMSC-CM than the medium with ASC-CM (Figure 5 and Figure 6). Therefore, the improvement in embryo developmental rate and antioxidant expression in BLs could be explained by the favorable culture conditions from the active antioxidants in AMSC-CM. 

In addition, apoptosis, which is generally accompanied by oxidative damage, was evaluated in BLs. The anti-apoptotic effect of AMSC-CM was confirmed in that pro-apoptotic gene expression was decreased and anti-apoptotic gene expression was increased. More specifically, an upregulation of the anti-apoptotic gene Bcl2 was observed not only in the AMSC-CM group, but also in the ASC-CM group, indicating that both CMs have anti-apoptotic effects. However, Caspase 3 which is known as an apoptosis executioner [67] was downregulated in the AMSC-CM group compared to the ASC-CM group (Figure 7). The caspase signaling pathway is activated by apoptosis-inducing factors released from the mitochondrial intermembrane space to the cytoplasm following the decrease of mitochondrial membrane potential, which is induced by oxidative damage in cells [68]. The effect of ASC-CM on mitochondrial membrane potential was not significant; however, as predicted, the ratio of JC-1 aggregate to JC-1 monomer was found to be higher in BLs cultured with AMSC-CM than in the control (Figure 8), indicating both anti-oxidative and anti-apoptotic effects of AMSC-CM on in vitro produced BL with enhanced mitochondrial membrane potential.

The present study compared antioxidant competence of CM obtained from two different types of MSC, ASC-CM and AMSC-CM, and suggested that AMSC-CM may be more efficient for embryo culture rather than ASC-CM. Our findings are supported by previous studies demonstrating that the quantity and variety of secretome from MSC can alter depending on different tissue sources of origin [29,69,70]. A point to be considered is that, to date, it is uncertain that MSC-CM can outperform chemical antioxidant compounds. To cite an example, several studies indicated that resveratrol, one of the chemically defined antioxidants which has been extensively studied [71], achieved less effective outcomes than MSC in pathological condition and diseases related to oxidative damage [72,73,74]. However, a direct comparison of MSC-CM and other antioxidant compounds has never been conducted to the best of our knowledge, especially in terms of assisted reproduction, and further studies are expected for clarification.

## 5. Conclusions

In conclusion, this study established that AMSC-CM treatment, at the optimal concentration, acts as an antioxidant during IVC of mouse preimplantation embryos. Furthermore, AMSC-CM treatment had a beneficial effect on embryo developmental rate and upregulated the FoxO-mediated expression of antioxidant enzymes in BLs cultured with AMSC-CM. Compared with ASC-CM, as a conventional antioxidant, AMSC-CM demonstrated enhanced expression of both enzymatic and non-enzymatic antioxidants, promotion of anti-oxidative culture conditions, and anti-apoptotic effects on developed embryos. These findings indicate that AMSC-CM can be developed as a novel and competent antioxidant interventions for the improvement of assisted reproductive technologies.

## Figures and Tables

**Table 1:** List of primer and sequence used for quantitative reverse transcription-polymerase chain reaction.

Gene	Accession No.	Primer Sequence
18S rRNA	NR_003278.3	F: ACCGCGGTTCTATTTTGTTG
		R: CCCTCTTAATCATGGCCTCA
AMPK	NM_001013367.3	F: GCTGTGGCTCACCCAATTAT
		R: ATCAAAAGGGAGGGTTCCAC
JNK	NM_016700.4	F: CGGAACACCTTGTCCTGAAT
		R: GAGTCAGCTGGGAAAAGCAC
AKT	NM_001165894.1	F: ACTCATTCCAGACCCACGAC
		R: GTCCAGGGCAGACACAATCT
SIRT1	NM_001159589.2	F: AGTTCCAGCCGTCTCTGTGT
		R: GATCCTTTGGATTCCTGCAA
FoxO1	NM_019739.3	F: ACATTTCGTCCTCGAACCAG
		R: CAGGTCATCCTGCTCTGTCA
FoxO3	NM_019740.3	F: ATGGGAGCTTGGAATGTGAC
		R: TTAAAATCCAACCCGTCAGC
SOD2	NM_013671.3	F: CTGTCTTCAGCCACACCAGA
		R: CTGCTCTTCCAAAGGTCCTG
Catalase	NM_009804.2	F: TTGACAGAGAGCGGATTCCT
		R: TCTGGTGATATCGTGGGTGA
GPx1	NM_008160.6	F: CCGACCCCAAGTACATCATT
		R: CCCACCAGGAACTTCTCAAA
Bax	NM_007527.3	F: ACCAAGAAGCTGAGCGAGTG
		R: TGCAGCTCCATATTGCTGTC
Bcl2	NM_009741.5	F: ATGATAACCGGGAGATCGTG
		R: AGCCCCTCTGTGACAGCTTA
Caspase3	NM_001284409.1	F: TGTCATCTCGCTCTGGTACG
		R: ATTTCAGGCCCATGAATGTC

F, Forward primer; R, Reverse primer.

**Table 2:** Effect of human amniotic membrane-derived mesenchymal stem cell conditioned medium (AMSC-CM) on in vitro fertilized mouse embryos development.

Group	No. of Cultured Embryos	No. of Embryos Developed to (%)			
		4-Cell	16-Cell	Blastocyst	Hatched Blastocyst
Control	72	66 (91.8 ± 1.8) ^b^	51 (71.1 ± 2.6) ^ab^	28 (38.6 ± 4.5) ^a^	14 (19.4 ± 4.6) ^a^
10% AMSC-CM	74	69 (92.5 ± 4.8) ^b^	55 (74.3 ± 4.8) ^b^	39 (51.7 ± 4.1) ^b^	25 (32.6 ± 5.9) ^b^
20% AMSC-CM	74	69 (93.4 ± 3.0) ^b^	45 (61.2 ± 3.5) ^a^	23 (30.7 ± 5.5) ^a^	15 (19.1 ± 3.5) ^a^
50% AMSC-CM	76	61 (79.6 ± 4.0) ^a^	46 (59.5 ± 3.8) ^a^	24 (28.3 ± 6.8) ^a^	16 (18.8 ± 3.4) ^a^

Experiments were repeated at least 3 times. ^a,b^ Mean ± SEM with different superscript letters indicate significant differences (at least *p* < 0.05).

**Table 3:** Effect of human adipose-derived mesenchymal stem cell conditioned medium (ASC-CM) and amniotic membrane-derived mesenchymal stem cell conditioned medium (AMSC-CM) on in vitro fertilized mouse embryos development.

Group	No. of Cultured Embryos	Number of Embryos Developed to (%)			
		4-Cell	16-Cell	Blastocyst	Hatched Blastocyst
Control	135	127 (93.1 ± 2.0)	101 (73.7 ± 3.3) ^a^	62 (44.4 ± 5.2) ^a^	40 (27.4 ± 8.0)
ASC-CM	134	124 (91.9 ± 1.7)	108 (79.2 ± 4.0) ^ab^	76 (56.4 ± 2.8) ^ab^	44 (32.2 ± 4.0)
AMSC-CM	130	125 (95.7 ± 1.4)	117 (87.6 ± 5.1) ^b^	85 (65.7 ± 3.3) ^b^	53 (39.7 ± 2.8)

Experiments were repeated at least 3 times. ^a,b^ Mean ± SEM with different superscript letters indicate significant differences (at least *p* < 0.05).

## Supplementary Materials

The following are available online at https://www.mdpi.com/2076-3921/10/2/268/s1, Table S1: Analysis of the quantity and quality of the extracted RNA.
