# Supplementary material for: Comparison of Anti-Oxidative Effect of Human Adipose- and Amniotic Membrane-Derived Mesenchymal Stem Cell Conditioned Medium on Mouse Preimplantation Embryo Development
Source: Antioxidants (Basel). 2021 Feb 9;10(2):268. doi: 10.3390/antiox10020268 (PMC7916131; doi:10.3390/antiox10020268)
Supplement: Supplementary file 1 [file antioxidants-10-00268-s001.pdf]

Table S1. Analysis of the quantity and quality of the extracted RNA.

Group	Concentration (ng/ul)	OD 260/280 ratio (nm)
Control	11.4	2.02
ASC-CM	12.1	1.92
AMSC-CM	13.0	2.01
